# ERAS protocol in laparoscopic surgery for colonic versus rectal carcinoma: are there differences in short-term outcomes?

**DOI:** 10.1007/s12032-016-0772-6

**Published:** 2016-05-06

**Authors:** Michał Pędziwiatr, Magdalena Pisarska, Michał Kisielewski, Piotr Major, Anna Mydlowska, Mateusz Rubinkiewicz, Marek Winiarski, Andrzej Budzyński

**Affiliations:** 2nd Department of General Surgery, Jagiellonian University Medical College, Kraków, Poland; Department of Endoscopic, Metabolic and Soft Tissue Tumors Surgery, Kopernika 21, 31-501 Kraków, Poland

**Keywords:** Fast-track surgery, Enhanced recovery after surgery, Laparoscopy, Colorectal cancer, Length of stay, Postoperative recovery

## Abstract

Most of the studies concerning enhanced recovery after surgery (ERAS) protocols in colorectal surgery include heterogeneous groups of patients undergoing open or laparoscopic surgery, both due to colonic and rectal cancer, thus creating a potential bias. The data investigating the differences between patients operated for either colonic or rectal cancer are sparse. The aim of the study was to compare short-term outcomes of laparoscopic surgery for colonic and rectal cancer with ERAS protocol. The analysis included consecutive prospectively registered patients operated for a colorectal cancer between January 2012 and September 2015. Patients were divided into two groups (colon vs. rectum). The measured outcomes were: length of stay (LOS), complication rate, readmission rate, compliance with ERAS protocol elements and recovery parameters (tolerance of early oral diet, mobilization and time to first flatus). Group 1 (colon) consisted of 150 patients and Group 2 (rectum) of 82 patients. Patients in Group 1 (150 patients) were discharged home earlier than in Group 2 (82 patients)—median LOS 4 versus 5 days, respectively. There was no statistical difference in complication rate (27.3 vs. 36.6 %) and readmissions (7.3 vs. 6.1 %). Compliance with the protocol was 86.9 and 82.6 %, respectively. However, in Group 1, the following procedures were used less frequently: bowel preparation (24 vs. 78.3 %) and postoperative drainage (23.3 vs. 71.0 %). There were no differences in recovery parameters between the groups. Univariate logistic regression showed that the type of surgery, drainage and stoma creation significantly prolonged LOS. In a multivariate logistic regression model, only a bowel preparation and drainage were shown to be significant. Although functional recovery and high compliance with ERAS protocol are possible irrespective of the type of surgery, laparoscopic rectal resections are associated with a longer LOS.

## Introduction

Recent studies, published within the last 10 years, concerning perioperative care in colorectal surgery, unequivocally recommend the introduction of perioperative care protocols based on the principles of enhanced recovery after surgery (ERAS) [1, 2]. Their implementation reduces complication rates, shortens the length of hospital stay (LOS) and accelerates postoperative recovery. Additionally, initial reports suggest modern perioperative care may influence long-term postoperative outcomes [3].

Most of the studies concerning ERAS protocols in colorectal surgery include heterogeneous groups of patients undergoing open or laparoscopic surgery, both due to colonic and rectal cancer, thus creating a potential bias [1, 2, 4]. The data investigating the differences between patients operated for either colonic or rectal cancer are sparse. Despite the fact most ERAS Society recommendations are similar regardless the type of surgery, there is a specific set of items concerning colonic and rectal resections. Moreover, the guidelines concerning these procedures are separate [5, 6]. Additionally, it has not been fully investigated, whether differences in recovery after laparoscopic surgery for colon or rectal cancer combined with the ERAS protocol exist. Previous analyses document a higher complication risk, longer LOS and slower recovery after open rectal comparing to colonic resection combined with traditional perioperative care [7].

The purpose of the study was to analyze the influence of the ERAS protocol implementation on short-term outcomes in laparoscopic colonic and rectal surgery for cancer.

## Methods

The prospective analysis included consecutive patients, who underwent laparoscopic colorectal cancer resection in the period from January 2012 to September 2015. The inclusion criteria for patients qualifying for the study consisted of: older than 18 years of age, an elective laparoscopic resection for histopathologically confirmed adenocarcinoma of the large intestine, perioperative care based on the ERAS protocol, which was the same irrespective of the type of the procedure (Table 1). The study excluded patients who were initially submitted for an open resection, emergency surgery, multivisceral resection, patients with rectal cancer treated with the use of transanal endoscopic microsurgery (TEM) and transanal total mesorectal excision (TaTME). Additionally, patients with coexisting inflammatory bowel diseases, and those in whom the fulfillment of the ERAS protocol was not possible (e.g., due to direct transfer to an intensive care unit immediately after the procedure), were also excluded.

**Table 1:** ERAS protocol used in our department

1. Preoperative counseling and patient’s education
2. No bowel preparation (oral lavage in the case of low rectal resection with TME and defunctioning loop ileostomy)
3. Preoperative carbohydrate loading (400 ml of Nutricia preOp^®^ 2 h prior surgery)
4. Antithrombotic prophylaxis (Clexane^®^ 40 mg sc. starting in the evening prior surgery)
5. Antibiotic prophylaxis (preoperative cefuroxime 1.5 g + metronidazole 0.5 g iv 30–60 min. prior surgery)
6. Laparoscopic surgery
7. Balanced intravenous fluid therapy (<2500 ml intravenous fluids during the day of surgery, <150 mmol sodium)
8. No nasogastric tubes postoperatively
9. No drains left routinely for colonic resections, one drain placed for <24 h in case of TME
10. TAP block and standard anesthesia protocol
11. Avoiding opioids, multimodal analgesia (oral when possible—paracetamol 4 × 1 g, ibuprofen 2 × 200 mg, metamizole 2 × 500 mg, or ketoprofen 2 × 100 mg)
12. Prevention of postoperative nausea and vomiting (PONV) (dexamethasone 8 mg iv., ondansetron 8 mg iv., metoclopramide 10 mg iv.)
13. Postoperative oxygenation therapy (4–6 l/min.)
14. Early oral feeding (oral nutritional supplement 4 h postoperatively—Nutricia Nutridrink^®^ or Nestlé Impact^®^, light hospital diet and oral nutritional supplements on the first postoperative day, full hospital diet in the second postoperative day)
15. Urinary catheter removal on the first postoperative day
16. Full mobilization on the first postoperative day (getting out of bed, going to toilette, walking along the corridor, at least 4 h out of bed)

For the purpose of further analysis, patients were divided into two groups, depending on the location of the tumor (and, as a result, the type of the performed operation). Group 1 included patients undergoing colonic resection. Group 2 consisted of patients with rectal cancer, where low anterior resection of the rectum (LAR) with/without defunctioning ileostomy or extralevator abdominoperineal resection was performed. Procedures were performed laparoscopically according to all oncological principles as described elsewhere [8].

The primary outcomes were as follows: length of stay (primary LOS, excluding readmissions), complication rate (according to Clavien–Dindo classification) and 30-day readmission rate [9]. The secondary outcome was compliance with the ERAS protocol. Compliance was analyzed in a similar manner to Gustafsson et al. [4] based on the presence or absence of 13 protocol elements in the pre- and intraoperative period, the application whereof depended on the consultant colorectal surgeon and the entire team involved in the perioperative treatment. The tertiary outcome was the assessment of the postoperative recovery, based on such protocol elements as: tolerance of oral diet on the first day after surgery (in all patients, oral diet was introduced in the evening on the day of the operation with fluids and oral nutritional supplements and was then extended to a full hospital diet on the first postoperative day), patients’ mobilization on the day of surgery (independent sitting up, a short walk to the toilet—all patients were actively mobilized by the nursing staff), time to first flatus and the necessity to administer opioids within the first 24 h after the procedure. The discharge criteria were: full mobilization, full tolerance of oral diet, no need for intravenous fluids or drugs and no complications. A surgeon examined every patient 5–7 days post-discharge in an outpatient clinic. Any hospitalization of the patient within 30 days post-surgery, after being discharged home, was considered a readmission.

The study has been approved by the Ethics Review Committee of the Jagiellonian University (approval number KBET/53/B/2014) and has been performed in accordance with the ethical standards laid down in the 1964 Declaration of Helsinki and its later amendments. All participants gave their informed consent in writing prior to inclusion in the study.

StatSoft STATISTICA version 10 was used for statistical analysis. For the purposes of further analysis, the entire group of patients was divided into 2 subgroups, depending on the location of the tumor (colon vs. rectum). The results are presented as mean ± standard deviation (SD), median and interquartile range (IQR) and odds ratio (OR) with 95 % confidence intervals (CI) when appropriate. The study of categorical variables used the Chi-square test of independence. Shapiro–Wilk test was used to check for normal distribution of data, and the student t test was used for normally distributed quantitative data. For non-normally distributed quantitative variables, the Mann–Whitney U test was used. Univariate logistic regression analysis of the individual demographic and perioperative parameters, that were significantly different between groups, was undertaken to assess the factors influencing prolonged LOS. Finally, factors significant in this univariate logistic regression analysis were used to build a multivariate logistic regression model. Results were considered statistically significant when *p* value was found to be <0.05.

## Results

**Fig. 1:**
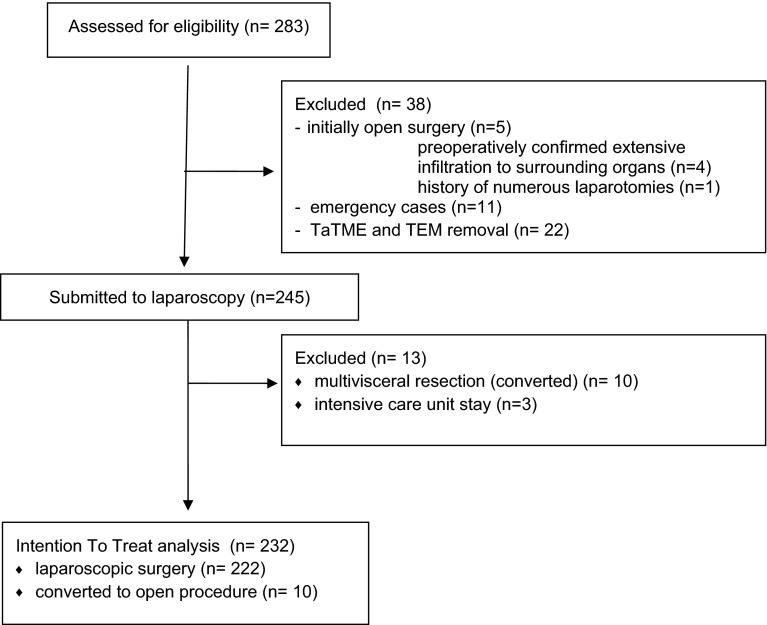
Patient flowchart

During the analyzed period, 283 patients with colorectal carcinoma were operated in our department. 38 patients were excluded from the study: 16 Patients underwent emergency surgery or were selected for an open surgery, and 22 patients with rectal cancer were operated with the use of TEM and TaTME methods. Additional 13 patients were excluded from the study: 3 patients due to the necessity to stay in the intensive care unit immediately after the operation and 10 patients due to multiple organ resections (Fig. 1).

After inclusion and exclusion criteria were met Group 1 (colon) consisted of 150 patients and Group 2 (rectum) consisted of 82 patients. Demographic characteristics and operative parameters in the studied groups are presented in Table 2. Patients from Group 1 were, on average, 3-year older than in group 2 (67.7 ± 13.2 vs. 64.1 ± 10.3 years, *p* = 0.0069). The differences in sex distribution between the two groups were also observed. In Group 1, there were more females than in Group 2 (52.0 vs. 34.1 %, *p* = 0.0086). There was no statistical difference between the two group based on other demographic parameters such as ASA scale, BMI and the stage of cancer.

**Table 2:** Demographic analysis of patient groups

Parameter	Group 1 (colon)	Group 2 (rectum)	*p* value
Number of patients (n)	150 (64.7 %)	82 (35.3 %)	–
Females [n (%)]	78 (52.0 %)	28 (34.1 %)	0.0086
Males [n (%)]	72 (48.0 %)	54 (65.9 %)	
Mean age (years ± SD)	67.7 ± 13.2	64.1 ± 10.3	0.0069
BMI (kg/m^2^ ± SD)	26.0 ± 5.1	26.8. ± 4.8	0.1489
ASA 1 [n (%)]	3 (2.0 %)	2 (2.4 %)	0.1903
ASA 2 [n (%)]	90 (60.0 %)	58 (70.7 %)	
ASA 3 [n (%)]	52 (34.7 %)	22 (26.9 %)	
ASA 4 [n (%)]	5 (3.3 %)	–	
AJCC stage I [n (%)]	47 (31.3 %)	30 (36.6 %)	0.2603
AJCC stage II [n (%)]	43 (28.7 %)	27 (32.9 %)	
AJCC stage III [n (%)]	43 (28.7 %)	14 (17.1 %)	
AJCC stage IV [n (%)]	17 (11.3 %)	11 (13.4 %)	
Right hemicolectomy [n (%)]	81 (54.0 %)	–	–
Left hemicolectomy [n (%)]	13 (8.7 %)	–	
Sigmoid resection [n (%)]	54 (36.0 %)	–	
Hartmann’s operation [n (%)]	2 (1.3 %)	–	
Low anterior resection of the rectum [n (%)]	–	76 (92.7 %)	
Abdominoperineal excision [n (%)]	–	6 (7.3 %)	
Formation of stoma	5 (3.3 %)	41 (50.0 %)	<0.0001
Colostomy	5 (3.3 %)	9 (11.0 %)	
Ileostomy	–	32 (39.0 %)	
Mean operative time (min. ± SD)	186.7 ± 57.7	200.5 ± 64.5	0.1080
Median operative time [min.(IQR)]	180 (140–212.5)	200 (150–240)	
Mean intraoperative blood loss (ml ± SD)	96.8 ± 83.3	110.7 ± 96.3	0.2640
Median intraoperative blood loss [ml (IQR)]	70 (50–150)	100 (50–150)	
Conversion [n (%)]	7 (4.7 %)	3 (3.7 %)	0.7145

The mean operative time in Group 1 was 186.7 ± 57.7 min. and in Groups 2—200.5 ± 64.5 min. (*p* = 0.1080), intraoperative blood loss was 96.8 ± 83.3 versus 110.7 ± 96.3 ml (*p* = 0.2640). Group 2 had significantly more patients with stoma (50 %, defunctioning ileostomy 32 cases and colostomy 9 cases). In Group 1 (3.3 %), the Hartmann procedure was performed in 2 patients and colostomy in 3 patients, in whom radical resection of the tumor was not possible.

**Fig. 2:**
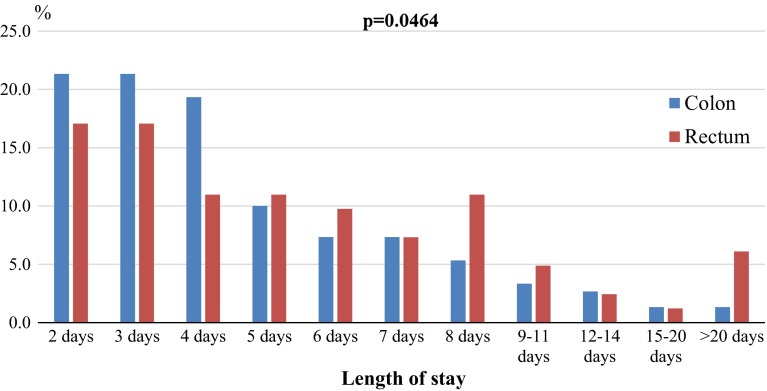
Percentage of patients based on the length of stay in hospital depending on the type of surgery

Primary outcomes: Patients undergoing laparoscopic surgery for colonic cancer were discharged home earlier, and then those treated for rectal cancer (Fig. 2). Median and mean LOS in Group 1 were 4 and 4.9 ± 3.8 days, whereas in Group 2 it was 5 and 6.5 ± 6.5 days (*p* = 0.0464).

There was no statistical difference in postoperative complications between the two groups (27.3 vs. 36.6 %, *p* = 0.1438). Table 3 details differences in types of observed complications between the two groups (the most severe complications in each patient are presented). The difference in severity of complications according to Clavien–Dindo classification was not significant (*p* = 0.5834). No differences were found in the 30-day readmission rates between the two groups (7.3 vs. 6.1 %, respectively, *p* = 0.7202).

**Table 3:** Types of complications according to Clavien–Dindo classification

Clavien–Dindo classification	Complications	Group 1 (colon)		Group 2 (rectum)		*p* value
V	Death (anastomotic leakage, reoperation, myocardial infarction during relaparotomy)	1	1 (0.7 %)	0	–	0.5834
IV	Anastomotic leakage (ICU stay)	1	1 (0.7 %)	0	–	
III B	Anastomotic leakage	2	7 (4.7 %)	1	8 (9.7 %)	
	Perforation of transverse colon from Veress needle	0		1		
	Perforation of small intestine	1		0		
	Peristomal fistula	0		1		
	Stoma necrosis	0		1		
	Trocar-related abdominal wall bleeding	1		0		
	Postoperative paralytic ileus	0		1		
	Cholecystitis	1		0		
III A	Anastomosis leakage (managed with Endo-SPONGE^®^)	0		3		
	Bleeding from anastomotic suture line (controlled endoscopically)	2		0		
II	Anastomotic leakage (confirmed in CT, managed conservatively)	1	7 (4.7 %)	0	3 (3.6 %)	
	Intraperitoneal hematoma	0		1		
	Urinary tract infection	2		1		
	Infectious diarrhea (C. difficile)	1		0		
	Pneumonia	1		0		
	Fever of unknown origin	1		0		
	Urinary retention	1		0		
	Perineal abscess after APR	0		1		
I	Surgical site infection	7	25 (16.7 %)	4	19 (23.2 %)	
	Postoperative nausea and vomiting	7		4		
	Non-infectious diarrhea	2		1		
	Postoperative paralytic ileus (managed conservatively)	2		5		
	High-output stoma	0		3		
	Bleeding from anastomosis suture line	2		1		
	Surgical site hematoma	3		1		
	Arrhythmia	1		0		
	Postoperative confusion	1		0		

**Fig. 3:**
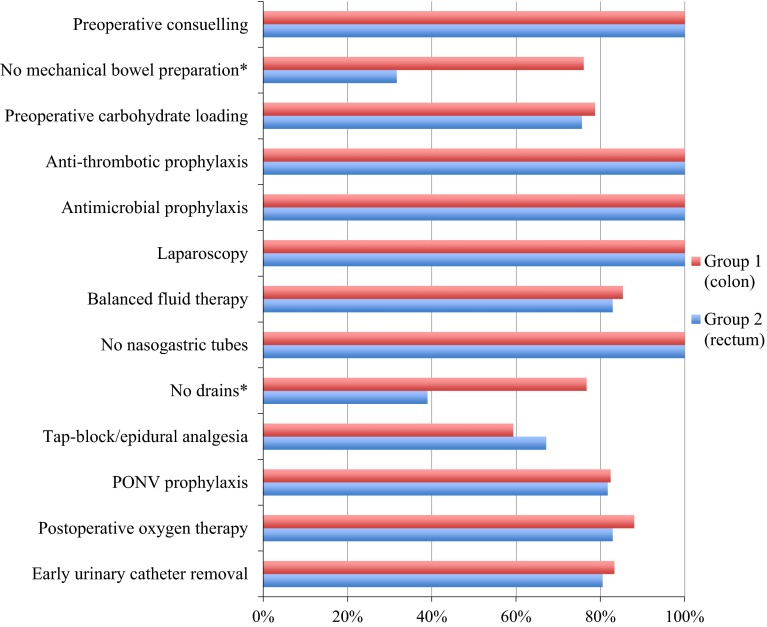
Compliance with perioperative elements of ERAS protocol in both groups. Statistically significant differences between the groups are marked with an asterisk. *Note*: In the case of MBP, the chart presents the actual percentage of patients receiving no MBP, not the compliance with the protocol (no MBP in the case of colonic surgery, oral bowel preparation in the case of LAR with ileostomy)

Secondary outcomes: Analysis of individual elements of perioperative care showed that compliance with pre- and intraoperative elements of the protocol was 86.9 ± 10.8 % and 82.6 ± 14.2 % in Group 1 and 2 (*p* = 0.0689). However, in Group 1, when compared to Group 2, the following procedures were used less frequently: MBP (24 vs. 78.3 %, *p* < 0.0001) and postoperative drainage (23.3 vs. 71.0 %, *p* < 0.0001). Figure 3 presents the compliance with each of the 13 elements of the ERAS protocol. In the case of MBP, the actual percentage of patients with no MBP is presented, not the compliance with the protocol (according to the guidelines, MBP is justified before rectal resection with defunctioning ileostomy).

Tertiary outcomes: No statistical differences in recovery parameters between the two groups were observed. The time to first flatus (days) was 1.8 ± 1.4 and 2.1 ± 1.9 in Group 1 and 2, respectively (*p* = 0.6408). No influence on the mobilization of a patient on the day of surgery depending on the type of the procedure performed was observed. In Group 1—136 patients (90.7 %) and in Group 2—68 (82.9 %) patients were ambulated (*p* = 0.08971). Full tolerance of an oral diet on the first postoperative day was observed in 115 (76.7 %) patients in Group 1 and 56 (68.3 %) in Group 2 (*p* = 0.1695). The need for opioids occurred in 48 (32 %) patients in Group 1 and in 35 (42.7 %) in Group 2 (*p* = 0.1063).

In the second stage of the analysis, the elements that varied between the groups and their relation with the LOS were taken into the consideration. The analysis of univariate logistic regression (the cutoff was the median LOS for all the patients—4 days) showed that the type of the surgery (OR 1.89, 95 % CI 1.10–3.27), MBP (OR 2.81 95 % CI 1.63–4.86), postoperative drainage (OR 3.42 95 % CI 1.95–5.99) and presence of stoma (OR 2.70 95 % CI 1.38–5.28) significantly prolonged LOS. Additionally, a multivariate logistic regression model was built, using elements that were significant in univariate regression analysis, and showed that MBP and postoperative drainage constitute a risk factor for prolonged LOS (Table 4).

**Table 4:** Uni- and multivariate logistic regression analysis of the parameters prolonging length of stay

Parameter	Univariate logistic regression			Multivariate logistic regression		
	OR	95 % CI	*p* value	OR	95 % CI	*p* value
Age (>65 vs. ≤65 years)	1.37	0.80–2.34	*p* = 0.2562	**–**		
Sex (male vs. female)	1.66	0.98–2.83	*p* = 0.0603	–		
BMI (>25 kg/m^2^ vs. ≤25 kg/m^2^)	1.04	0.67–1.62	*p* = 0.8634	–		
ASA grade (4–1)	1.06	0.67–1.67	*p* = 0.8149	–		
AJCC stage (IV—I)	1.09	0.85–1.41	*p* = 0.4894	–		
Mechanical bowel preparation (yes vs. no)	2.81	1.63–4.86	*p* = 0.0002	2.24	1.19–4.20	*p* = 0. 0123
Peritoneal drainage (yes vs. no)	3.42	1.95–5.99	*p* = 0.00002	2.85	1.54–5.28	*p* = 0.0009
Stoma formation (yes vs. no)	2.70	1.38–5.28	*p* = 0.0039	1.54	0.65–3.63	*p* = 0.3207
Rectum/colon	1.89	1.10–3.27	*p* = 0.0231	1.34	0.63–2.85	*p* = 0.4506

## Discussion

This study of patients after laparoscopic operations, in whom the ERAS protocol was applied, showed that the length of stay after a colonic resection was significantly shorter compared to rectal surgery, despite a lack of differences in compliance with the protocol, functional recovery time and the complication rate. It was found, however, that MBP, postoperative drainage and presence of stoma may affect LOS.

Although in most of publications concerning colorectal surgery patients with colonic or rectal carcinoma are analyzed together as one group, some differences between the outcomes may usually be found. Faiz et al. [7] having analyzed over 180 thousand patients after open resections found that the type of procedure significantly influences LOS—operations involving distal parts of the large bowel were associated with a longer LOS. A MRC CLSICC trial showed similar observations and additionally found that using laparoscopic approach reduces LOS [10]. Influence of laparoscopic surgery on LOS was also showed in other randomized studies [11–13]. In our analysis of patients operated laparoscopically, this difference between colonic and rectal operations still occurs. However, thanks to the application of the ERAS protocol, LOS may be shortened even further as it has been shown in other studies [14, 15]. Although there is evidence that rectal surgery may be connected with a higher complication rate, no such observation was noted among our patients; therefore, prolonged LOS seen in this publication cannot be solely explained this way [16].

The correlation between the compliance with the protocol and short-term outcomes, including LOS, is commonly known [16–18]. However, the application of all elements of the protocol is possible only in a low percentage of patients, and most of the authors present the compliance level of 75–85 % [4, 16, 18]. The ongoing discussion on the validity of the application of all elements suggested in the ERAS guidelines continues, since it is not known which ones have the biggest influence on the patients’ recovery. In this study, there was no difference in regard to total adherence to the protocol between patients with rectal and colonic cancer. This observation is different from the data published by the ERAS Compliance Study Group [16]. However, essential differences in perioperative procedures were observed in relation to two elements of the protocol—mechanical bowel preparation and postoperative drainage. For a long time, both these elements constituted dogmas in perioperative care in colorectal surgery. Despite existing evidence justifying the omission of MBP, it is still performed routinely in many countries [19–21]. In our protocol, according to ERAS guidelines, oral bowel preparation was allowed in the case of LAR with defunctioning ileostomy (therefore, in these cases this element did not lower total compliance). Our data showed that a longer LOS was observed in patients from Group 2 in whom MBP was performed more commonly. Other studies confirm this and suggest that it may even increase the risk of non-surgical complications [22, 23]. In contrast, recent publications indicated that MBO together with oral antibiotics may indeed lower surgical site infection and leakage rate [24–26]. This definitely gives new insight into the problem and therefore requires further investigations. Moreover, it is still not uniformly defined, whether the large bowel should be cleaned before performing a rectal resection with defunctioning ileostomy. Common sense (as well as ERAS guidelines) suggests that if we want to divert the intestinal passage from the anastomosis, MBP may be important. Some authors say it is not necessary because MBP does not lower the rate of leakage from the anastomosis [27]. Similarly, use of postoperative drainage does not lower the leakage rate and may lead to prolonged hospitalization [28]. In our department, a drain after a non-complicated resection is left for 24 h only in the case of LAR and we acknowledge this is a divergence from ERAS guidelines. The decision of removal of drainage is made by consultant surgeon based on postoperative presentation of patient. We observed that in a significant percentage of patients, the drain is left for a longer period of time which might be influenced by a deep-rooted habit inherited from the times of traditional care, when drain was kept until the drainage decreased significantly. This habit undoubtedly influenced the compliance in the group of patients with rectal carcinoma and increased the LOS among those patients.

In this study, there was a higher percentage of stoma in the group of patients treated for rectal cancer when comparing to colonic (41 vs. 3.3 %, respectively). According to Faiz et al. [7] defunctioning ileostomy (in our study—elective procedure in the case of LAR) prolongs hospitalization, even though the small bowel can, theoretically, recover faster than the large bowel. A univariate logistic regression analysis of our patients shows significantly prolonged hospital stay among patients with stoma; however, a multivariate analysis did not confirm these results, which may mean there are other, stronger factors influencing LOS which requires further studies. One of the possible explanations for a prolonged hospital stay among patients with stoma is that they require a training and explanation of how to take care of it, which, with a very short stays lasting for 2–3 days may be impossible. Such patients, in spite of meeting the objective discharge criteria, may require to stay longer in the hospital to receive more education before being discharged home. Therefore, some authors suggest that such training should be started prior to surgery or even before admission to a hospital [29, 30]. Another explanation for prolonged LOS among patients with stoma was a significantly higher percentage of patients who suffered from problems with emptying on the first postoperative day and symptoms of postoperative paralytic ileus. Also, several patients presented symptoms of a high-output stoma, which required adjustment and additional days of hospitalization. Although, according to the Clavien–Dindo classification, these are low-grade complications, and their occurrence was associated with a longer hospital stay. Nonetheless, for some time now, defunctioning ileostomy in patients after a low anterior rectal resection is performed routinely in our unit (approximately from halfway into the time of the study), since, according to the latest meta-analyses, it decreases the anastomotic leakage rate, rate of severe complications and need for reoperation, which fully justifies such a procedure [31, 32].

In the questionnaire conducted during the first ERAS Congress in Cannes in 2013, respondents have clearly stated that, currently, the main aim in perioperative care is not the length of hospital stay, but full functional recovery after surgery [33]. In our study, we did not observe the postoperative parameters as mobilization on the day of the procedure, return of the gastrointestinal tract functions (time to first flatus) and tolerance of oral diet on the first postoperative day varied between the groups. No differences in demand for opioids were found, either. Therefore, we can conclude that the recovery on the first days after the procedure measured with these parameters is similar and there are factors other than the clinical condition of the patient (drainage, bowel preparation, stoma), which prolong LOS. It is an important observation in the pending discussion on the necessity of application of all of the elements of the protocol in the perioperative period and suggests necessity of further studies of this subject.

## Conclusions

Our observations confirm that laparoscopic rectal cancer operations are associated with longer hospital stays when compared to colonic resections, even with the implementation of the ERAS protocol. Reaching high ERAS protocol compliance and functional recovery on the same level is possible irrespective of the type of the procedure. However, other factors delaying discharge from a hospital have been identified: bowel preparation, postoperative drainage and defunctioning ileostomy. Therefore, the possible influence of those factors on postoperative outcomes should be emphasized and addressed in further studies.
